# Chromosomal microarray analysis as a first-tier clinical diagnostic test: Estonian experience

**DOI:** 10.1002/mgg3.57

**Published:** 2014-01-09

**Authors:** Olga Žilina, Rita Teek, Pille Tammur, Kati Kuuse, Maria Yakoreva, Eve Vaidla, Triin Mölter-Väär, Tiia Reimand, Ants Kurg, Katrin Õunap

**Affiliations:** 1 Department of Genetics, United Laboratories, Tartu University HospitalTartu, Estonia; 2 Department of Biotechnology, Institute of Molecular and Cell Biology, University of TartuTartu, Estonia; 3 Department of Pediatrics, University of TartuTartu, Estonia; 4 Department of Biomedicine, Institute of Biomedicine and Translation Medicine, University of TartuTartu, Estonia

**Keywords:** Chromosomal microarray analysis, copy-number variation, developmental delay, first-tier testing, intellectual disability, postnatal diagnosis, prenatal diagnosis

## Abstract

Chromosomal microarray analysis (CMA) is now established as the first-tier cytogenetic diagnostic test for fast and accurate detection of chromosomal abnormalities in patients with developmental delay/intellectual disability (DD/ID), multiple congenital anomalies (MCA), and autism spectrum disorders (ASD). We present our experience with using CMA for postnatal and prenatal diagnosis in Estonian patients during 2009–2012. Since 2011, CMA is on the official service list of the Estonian Health Insurance Fund and is performed as the first-tier cytogenetic test for patients with DD/ID, MCA or ASD. A total of 1191 patients were analyzed, including postnatal (1072 [90%] patients and 59 [5%] family members) and prenatal referrals (60 [5%] fetuses). Abnormal results were reported in 298 (25%) patients, with a total of 351 findings (1–3 per individual): 147 (42%) deletions, 106 (30%) duplications, 89 (25%) long contiguous stretches of homozygosity (LCSH) events (>5 Mb), and nine (3%) aneuploidies. Of all findings, 143 (41%) were defined as pathogenic or likely pathogenic; for another 143 findings (41%), most of which were LCSH, the clinical significance remained unknown, while 61 (18%) reported findings can now be reclassified as benign or likely benign. Clinically relevant findings were detected in 126 (11%) patients. However, the proportion of variants of unknown clinical significance was quite high (41% of all findings). It seems that our ability to detect chromosomal abnormalities has far outpaced our ability to understand their role in disease. Thus, the interpretation of CMA findings remains a rather difficult task requiring a close collaboration between clinicians and cytogeneticists.

## Introduction

DNA copy-number variations (CNVs) are widely recognized as a cause of genetic variation that could predispose to common and complex disorders, including developmental delay/intellectual disability (DD/ID), multiple congenital anomalies (MCA), and autism spectrum disorders (ASD) (Stankiewicz and Lupski 2010; Vissers et al. 2010; Coughlin et al. 2012). Having a high prevalence in the general population (DD/ID: 2–3%; ASD: ∼1:150 individuals), this category of disorders accounts for the largest proportion of cytogenetic testing (Miller et al. 2010). Chromosomal microarray analysis (CMA) offers the capacity to examine the whole human genome on a single chip with a resolution which is at least 10-fold greater than the best G-banded chromosome analysis, and is now established as the first-tier cytogenetic diagnostic test for fast and accurate detection of chromosomal abnormalities in this patient population (Miller et al. 2010). The decision to replace the traditional G-banding with the novel CMA was made based on the comparison of diagnostic yields of two techniques and the total cost of the analyses per patient. G-banded karyotyping alone detects pathogenic genomic imbalances in ∼3% of those patients (excluding clinically recognizable chromosomal syndromes, e.g., Down syndrome), whereas the diagnostic yield for CMA is 10–25% depending on the microarray platform and patient selection (Miller et al. 2010; Vissers et al. 2010; Ahn et al. 2013). In general, the adoption of microarrays for analysis of DNA copy-number changes by research and clinical diagnostic laboratories had a great impact on the field of medical genetics, enabling to clarify genotype–phenotype relationships in known disorders and to identify novel syndromes (Bejjani and Shaffer 2008; Coughlin et al. 2012).

In Estonia, CMA was introduced into clinical practice in 2009 and was performed in patients whose diagnosis remained unknown despite all routine genetic investigations. Since 2011, CMA is on the official service list of the Estonian Health Insurance Fund and is performed as the first-tier cytogenetic diagnostic test for patients with DD/ID, ASD, and/or MCA. Here, we present our experience with using CMA for postnatal and prenatal diagnosis in Estonian patients during 2009–2012.

## Materials and Methods

### Patients and samples

All samples in this study were received between January 2009 and December 2012, a total of 1191 patients (male/female ratio 58/42), including postnatal (1072 [90%] patients and 59 [5%] family members) and prenatal referrals (60 [5%] fetuses). The median age was 5 years (range: newborn to 83 years). The patient population sent for CMA before 2011 (a total of 188 individuals) was very carefully selected, and consisted of patients with an unknown diagnosis despite all routine genetic investigations. Since 2011, the cost of CMA is covered by the Estonian Health Insurance Fund, and the analysis is performed as the first-line cytogenetic diagnostic test in patients with DD/ID, ASD, and/or MCA as recommended by the International Standard Cytogenomic Array Consortium (Miller et al. 2010). For prenatal CMA testing, main indications were abnormal ultrasound findings, a positive aneuploidy screening result, family history of chromosomal abnormalities, and other exceptional conditions (e.g., repeated miscarriages and complicated anamnesis).

### CMA and interpretation

In case of postnatal testing, genomic DNA was extracted from peripheral blood according to the standard salting out protocol. For prenatal tests, the DNA extracted from amniotic fluid, chorionic villi or cultured cells was used. Only fetal samples that passed the maternal contamination test were analyzed. Screening for chromosomal rearrangements was performed using HumanCNV370-Quad or HumanCytoSNP-12 BeadChips (Illumina Inc., San Diego, CA), allowing the effective resolution of 49 and 62 kb, respectively (10 consecutive single-nucleotide polymorphism (SNP) markers). The genotyping procedures were performed according to the manufacturer's protocol. Genotypes were called by BeadStudio v.3.1 or GenomeStudio v2009.1 software (Illumina Inc.), and further CNV analysis and breakpoint mapping was conducted with QuantiSNP v1.1 or v2.1 software (Colella et al. 2007). Only samples with a call rate >98% that passed the QuantiSNP quality control parameters were analyzed. In mosaic cases, the level of mosaicism was determined based upon visual estimation of allele peak distribution pattern (Conlin et al. 2010). Fluorescence in situ hybridization (FISH), quantitative polymerase chain reaction (qPCR), G-banding or multiplex ligation-dependent probe amplification (MLPA) were used for confirmation studies. Inheritance pattern was examined either by CMA or other methods.

All detected copy-number changes were compared with known CNVs listed in the database of genomic variants (DGV) (Iafrate et al. 2004) and studied for genomic content using UCSC genome browser or ENSEMBL. Potential clinical significance of CNVs not present in normal individuals was estimated using DECIPHER and OMIM databases, and peer-reviewed literature searches in the PubMed database (Firth et al. 2009). A chromosomal aberration was defined as pathogenic or likely pathogenic if it (1) overlapped with a genomic region associated with a well-established syndrome, (2) was large in size (>5 Mb) containing a rich gene content, (3) or contained a gene or a part of a gene implicated in a known disorder (Miller et al. 2010). The CMA finding was considered as benign or likely benign if it (1) was present in healthy individuals (e.g., healthy family members [with some exceptions] or DGV), (2) was gene-poor and did not encompass any known disease-causing genes, (3) had not been previously reported in association with any disorders. All remaining findings were categorized as variants of uncertain clinical significance (VUCS).

## Results

During 4 years – from January 2009 until December 2012 – a total of 1191 CMA tests were ordered in Tartu University Hospital, and in 1003 cases, CMA was used as the first-line cytogenetic test. Ninety percent of referrals comprised of postnatal patients, 5% were family members, and the remaining 5% were prenatal analyses. The overall success rate was 99.5%. A repeat analysis was needed in six cases: five did not pass the quality control, and in one mosaic uniparental disomy (UPD) case, adjustment analysis was needed. Abnormal results were reported in 25% (298) of patients, altogether 351 findings (1–3 per individual, with a size range from tens of kb to entire chromosomes): 42% (147) were deletions, 30% (106) duplications, 25% (89) long contiguous stretches of homozygosity (LCSH) events (>5 Mb), and in 3% (9), an aneuploidy was detected. Among them, mosaicism was found in 2% (6) of the patients. If the two time periods are examined separately – 2009–2010, when CMA was applied only for patients whose routine genetic investigations did not give any results, and 2011–2012, when CMA became the first-tier cytogenetic test for patients with DD/ID, ASD or MCA – a difference in number of abnormal results can be observed: 32% and 24%, respectively. Over 80% of the detected CNVs (not including regions of LCSH) were <5 Mb and would likely be missed by traditional karyotyping; 39% were <1 Mb (deletions/duplications ratio 56/44). Based on the aforementioned criteria, 143 (41%) of the 351 findings in 126 patients were defined as pathogenic or likely pathogenic; for 143 (41%), most of which were LCSH, the clinical significance remained unknown; 61 (18%) of the reported findings could now be reclassified as benign or likely benign due to the advances in the field of molecular clinical genetics and addition of new entries to the publicly available databases. This means that clinically relevant findings were detected in 11% of all analyzed patients. The diagnostic yields for 2009–2010 and 2011–2012 periods were 15% and 10%, respectively. For cases with completed inheritance studies, 22% of imbalances were de novo. Confirmation studies using independent methods, such as qPCR, FISH, karyotyping, or MLPA, were performed for more than half of CNVs (148 of 262) and showed that four detected CNVs (10–970 kb) actually represented false-positive results. It cannot be excluded that there might be more false positives in our patient group.

### Pathogenic or likely pathogenic findings

Altogether 106 (30%) aberrations associated with known microdeletion and microduplication syndromes, or deletions encompassing a gene or a part of a gene implicated in human disease (most of those were <1 Mb) were detected (Table 1). Most frequent genomic disorders found in our dataset were 15q13.3 microdeletion/microduplication syndrome (nine cases), 15q11.2 microdeletion (seven cases), 16p11.2 microdeletion/microduplication syndrome (five cases), 1p36 microdeletion syndrome (four cases), Silver–Russell/Beckwith–Wiedemann syndrome (four cases, including one case of 11p15.5-15.4 UPD), Prader–Willi/Angelman syndrome (four cases, including one case of maternal UPD 15). Also, a relatively large number of aberrations in the recurrent microdeletion/microduplication loci with well-established association with abnormal phenotypes but with incomplete penetrance and variable expressivity were discovered, for example, 1q21.1 deletions/duplications (four cases) responsible for increased susceptibility to neurodevelopmental disorders, and 16p13.1 deletions/duplications (six cases) implicated in increased susceptibility to neurocognitive disorders (Ullmann et al. 2007; Mefford et al. 2008). Remarkably, all detected 1q21.1 aberrations were inherited; the inheritance studies for 16p13.1 imbalances have not been performed.

**Table 1:** Aberrations that overlap with critical genomic regions for microdeletion and microduplication syndromes, or encompass genes implicated in human diseases.

Cytoband	Syndrome/Disease	OMIM No.	Gene(s)	No. of deletion cases	No. of duplication cases
1p36	1p36 microdeletion	607872		4	–
1q21.1	1q21.1 deletion/duplication^1^	612474/612475	Contiguous gene deletion syndrome, incl. *GJA5*	2	2
1q43-q44	Megalencephaly-polymicrogyria-polydactyly-hydrocephalus syndrome	603387	*AKT3*	1	–
2p16.3	2p16.3 deletion	614332	*NRXN1*	1	–
2qq11.2	2q11.2 microdeletion	–	*LMAN2L*,*ARID5A*	1	–
2q31.2	2q31.2 deletion	612345	Contiguous gene deletion syndrome	1	–
2q37	2q37 microdeletion	600430	Contiguous gene deletion syndrome	1	–
3p25-pter	Distal 3p deletion	613792	Contiguous gene deletion syndrome	1	–
3p25.3	Von Hippel–Lindau syndrome	193300	*VHL*	1	–
3p13-p14	Waardenburg syndrome	193510	*MITF*	1	–
3q22.3	Blepharophimosis-ptosis-epicanthus inversus syndrome	110100	*FOXL2*	1	1
4p16.3	Wolf–Hirschhorn syndrome	194190	Contiguous gene deletion syndrome	1	2
4q22.1	Parkinson disease	168601	*SNCA*	–	2
5p15.2	Cri-du-Chat syndrome	123450	Contiguous gene deletion syndrome, incl. *TERT*	2	–
5p15.2	Mental retardation in Cri-du-Chat syndrome	123450	*CTNND2*	1	–
5q35.2-q35.3	Sotos syndrome/5q35 microduplication	117550/–	*NSD1*	2^2^	1
6q25.1-q25.2	Emery–Dreifuss muscular dystrophy 4, autosomal dominant	612998	SYNE1	1	–
7p21.1	Saethre–Chotzen syndrome	101400	*TWIST1*	1	–
7p14.1	Greig cephalopolysyndactyly syndrome/Pallister–Hall syndrome	175700/146510	GLI3	1	–
7q11.23	Williams–Beuren syndrome	609757	Contiguous gene deletion syndrome, incl. *ELN*	1	–
7q21.2-q21.3	Split-hand/foot malformation 1 with sensorineural hearing loss	220600	*DLX5*	1	–
7q36.3	Polydactyly, preaxial II	174500	*LMBR1*	1	–
8q24.13	Spastic paraplegia 8, autosomal dominant	603563	*KIAA0196*	1	–
10q23	Juvenile polyposis syndrome + 10q23 deletion	174900/612242	*NRG3*,*GRID1*,*PTEN*,*BMPR1A*	1	–
10q26	10q26 deletion	609625	Contiguous gene deletion syndrome	1	1
11p15.5	Beckwith–Wiedemann/Silver–Russell syndrome	130650/180860	Contiguous gene deletion syndrome, incl. *CDKN1C*,*H19*,*LIT1*	3	1
11q23	Jacobsen syndrome/Thrombocytopenia, Paris-Trousseau type	147791/188025	Contiguous gene deletion syndrome	1	–
12p12.1	DD, language delay, behavioral problems	–	*SOX5*	1	–
15q11.2^4^	Prader–Willi/Angelman syndrome (Type 1)	176270/105830	*NDN*,*SNRPN*,*UBE3A*	1	1
15q11.2	Prader–Willi syndrome/Angelman syndrome (Type 2)	176270/105830	*NDN*,*SNRPN*,*UBE3A*	1	–
15q11.2	15q11.2 microdeletion/microduplication^1^			6	1
15q13.3	15q13.3 microdeletion/microduplication^3^	612001	Contiguous gene deletion syndrome, incl. *CHRNA7*	7	2
16p11.2	16p11.2 microdeletion/microduplication	611913/614671	Contiguous gene deletion syndrome	4	1
16p12.1	16p12.1 microdeletion^1^	136570	Contiguous gene deletion syndrome	2	–
16p13.11	16p13.11 microdeletion/microduplication^1^	–	Contiguous gene deletion syndrome, incl. *MYH11*	1	5
16p13.2	Epilepsy with neurodevelopmental defects	613971	*GRIN2A*	1	–
17p13.3	17p13.3 distal deletion	–	*YWHAE*	2	–
17p12	Hereditary neuropathy with liability to pressure palsies	162500	*PMP22*	2	–
17p11.2	Smith–Magenis syndrome	182290	*RAI1*	1	–
17q11.2	Neurofibromatosis I	162200	*NF1*	2	–
17q21.31	Koolen-De Vries syndrome	610443	Contiguous gene deletion/duplication syndrome, incl. *MAPT*	1	–
18p	Chromosome 18p deletion syndrome	146390	Contiguous gene deletion syndrome	1	–
18p11.31	Holoprosencephaly 4	142946	*TGIF*	2	–
18q22.3-q23	Congenital aural atresia	607842	*TSHZ1*	1	–
22q11.2	DiGeorge/Velocardiofacial/Chromosome 22q11.2 duplication syndrome	188400/192430/608363	Contiguous gene deletion syndrome, incl. *TBX1* and *COMT*	3	1
22q13	Phelan–McDermid syndrome	606232	Contiguous gene deletion/duplication syndrome, incl. *SHANK3*	3	–
Xp22.31	Ichthyosis	308100	*STS*	1	–
Xp21.3-p21.2	X-linked mental retardation	300143	*IL1RAPL1*	3	–
Xp21.1	Duchenne muscular dystrophy	310200	*DMD*	3	–
Xq28	Rett syndrome	312750	*MECP2*	–	1
Yq11.21-q11.23	Spermatogenic failure	415000	*USP9Y*,*DB9*	1	–

1 Susceptibility locus.

2 One of the patients with a deletion of exons 3-8 of *NSD1* did not display a clinical phenotype of Sotos syndrome, but rather a phenotype of 5q35 microduplication.

3 Duplication represents a susceptibility locus.

4 In one case, maternal UPD was diagnosed.

Aneuploidies were discovered in eight (2%) patients (one trisomy 13, two monosomies X, two triple X syndromes, one Klinefelter syndrome, two XYY syndromes), which shows that aneuploidies are sometimes not easily recognizable on clinical ground.

Multiple LCSH distributed across the entire genome that obviously influence the phenotype by unmasking recessive mutations in disease-causing genes were observed in four cases (the percentage of genome that is identical by descent [IBD] varied from 4% to 22%), including two fetuses. Also, four cases of UPD associated with patients’ clinical phenotypes were found, including three mosaic cases: 4q31.3-q35.2 (50%), 11p15.5-p15.4 (50%) – Beckwith–Wiedemann syndrome, UPD 14, and maternal UPD 15 (50%) – Prader–Willi syndrome.

One approximately 45 kb size deletion in 2q33.1 reported as likely pathogenic was found to be a false-positive finding.

The remaining 24 aberrations classified as pathogenic or likely pathogenic did not overlap with any known syndrome, but were large in size (at least several Mb) and in gene-rich areas, which gives a reason to assume that they could be responsible for abnormal phenotypes.

### Variants of uncertain clinical significance

The clinical relevance of 143 (41%) reported findings remained unclear, altogether 64 deletions/duplications and 80 regions of LCSH. Most of the imbalances were <1 Mb and have not been previously implicated in human diseases. In about half of the deletion/duplication cases, inheritance studies were conducted, whereas only three imbalances appeared to be de novo. Still, the pathogenicity of inherited CNVs cannot be excluded before more information on those genome regions is available.

According to the laboratory policy, stretches of homozygosity larger than 5 Mb were reported. However, in most cases this turned out to be diagnostically unhelpful, as the vast majority of reported LCSH were classified as VUCS. The most promising finding was a 12 Mb homozygosity stretch in 3q13.13-q21.1 encompassing the *CASR* gene implicated in epilepsy, which correlates well with the patient's phenotype (Kapoor et al. 2008). However, Sanger sequencing of *CASR* has not been performed yet.

### Prenatal diagnosis

CMA with fetal DNA was performed in 60 cases, eight of which were ordered after the termination of the pregnancy. Indications for prenatal CMA testing are presented in Table 2. Array analysis was mostly performed simultaneously with karyotyping in order to enable better characterization of potential CMA findings and to detect aberrations that would be missed using CMA. In eight cases, an abnormal result was reported (Table 3).

**Table 2:** Prenatal CMA testing in Estonia during 2009–2012 (including fetuses analyzed after the termination of pregnancy).

Indication for prenatal diagnosis	Number of cases (%)
Familial balanced rearrangement	18 (30)
Anomaly on ultrasonography	13 (22)
Termination of pregnancy due to abnormal fetus	8 (13)
Positive triple test	5 (8)
Isolated abnormal nuchal translucency	5 (8)
Other child(ren) with chromosomal disease	4 (7)
Other child or parent with unspecified genetic pathology	3 (5)
Unspecified	3 (5)
Recurrent spontaneous abortions	1 (2)
Total	60 (100)

**Table 3:** CMA findings in prenatal tests (including cases tested after the termination of pregnancy).

Case	Indication	Karyotype	CMA	Clinical significance	Outcome
1	Isolated increased nuchal translucency	–	arr[hg19] Xp21.1(31,665,779–32,096,779) × 3	UCS	Termination of pregnancy
2	Recurrent spontaneous abortions	–	arr[hg19] 8q11.1q11.23(47,060,977–52,693,165) × 2 hmz	UCS	Tested after the termination of pregnancy
3	Positive triple test	46,XX	Multiple long stretches of homozygosity	Pathogenic	Termination of pregnancy
4	Positive triple test	46,XX[64]/47,XX,+7[9]	arr(7) × 2–3 (10–20%)	Likely benign	Normal female at term
5	Abnormal ultrasound	–	arr[hg19] 7p14.1p13(42,179,377–44,932,538) × 1	Pathogenic (Greig syndrome, OMIM 175700)	Tested after the termination of pregnancy
6	Familial balanced rearrangement	46,XX,rec(4)dup(4p)del(4q)inv(4)(p15.3q35)pat	arr[hg19] 4p13.33p16.3(1–13,912,694)x3, 4q35.2(188,730,709–190,880,409) × 1	Pathogenic	Termination of pregnancy
7	Familial balanced rearrangement	46,XY,inv(10)(p11.2;q21.2)mat	arr[hg19] 12q14.2q15(63,291,364–68,794,078) × 2 hmz	UCS	Not known
8	Dysmorphic fetus	–	Multiple long stretches of homozygosity	Pathogenic	Tested after the termination of pregnancy

CMA, chromosomal microarray analysis; UCS, unknown clinical significance.

In case 1, a duplication encompassing exons 45–51 of the *DMD* gene was detected in a male fetus (46,XY) and was confirmed by MLPA analysis using the SALSA MLPA P034-A2 and P035-A2 probe mix (MRC-Holland, The Netherlands). The mother did not carry the duplication and the pregnancy was terminated after counseling; however, later it was found that the father was a carrier of Xp21.1 duplication. Because chromosome X cannot be transferred to the male offspring through paternal line, the duplicated segment is likely to be inserted into some other chromosome. This theory has not been controlled though.

The indication for CMA in case 2 was recurrent spontaneous abortions of unknown etiology in the family. The analysis performed after the termination of the pregnancy revealed a 5.6 Mb LCSH on chromosome 8; however, its association with clinical problems remained uncertain.

In two cases (3 and 8), multiple regions of LCSH distributed across the entire fetal genome were discovered (the percentage of genome that is IBD was 6% and 20%, respectively).

In case 4, a low-level mosaic trisomy 7 (∼13% and ∼10%, respectively) was detected by G-banding and CMA using amniotic fluid cell culture. Although most cases with this chromosomal abnormality have no or only subtle clinical symptoms, a maternal UPD 7 strongly associated with severe growth restriction could not be excluded. Because some symptoms were observable on ultrasonography, additional amniocentesis was performed. FISH analysis showed the presence of additional chromosome 7 in 5% of the cells, while G-banding revealed a normal karyotype. However, a normal female was born at term with normal birth weight and length.

In case 5, an approximately 3 Mb deletion in 7p14.1-p13 was found, disrupting the *GLI3* gene associated with Greig cephalopolysyndactyly syndrome (OMIM 175700), which was concordant with the fetal dysmorphic phenotype.

Cases 6 and 7 were referred due to familial balanced rearrangements. In case 6, a terminal duplication of 4p (14 Mb) and terminal deletion of 4q (2 Mb) were detected, which were treated as pathogenic due to their size, and pregnancy was terminated. In case 7, the fetus was found to inherit an inv(10)(p11.2q21.2) from his mother, and no CNVs in inversion adjacent regions or elsewhere in the genome were detected by CMA. However, a 5.5 Mb LCSH with unclear clinical relevance was identified. The outcome of this pregnancy is not known.

## Discussion

Making the correct diagnosis in patients with DD/ID, ASD, and/or MCA is crucial for predicting the clinical progress with relative certainty, estimating the recurrent risk in a family, or simply bringing emotional relief to parents. Implementation of microarrays in clinical practice enabled to improve the diagnostic yield up to 10–25% in this patient group compared with 5–6% detected previously by karyotyping and subtelomeric FISH. In the context of prenatal diagnostic testing, CMA provided better detection of genetic abnormalities and identified additional, clinically significant cytogenetic information as compared with karyotyping and was equally efficacious in identifying aneuploidies and unbalanced rearrangements, but did not identify balanced translocations and triploidies (Reddy et al. 2012; Wapner et al. 2012). Therefore, currently CMA is recommended as the first-tier diagnostic test for patients with DD/ID, ASD, and/or MCA (Miller et al. 2010). Our clinical experience shows similar results. Nevertheless, the interpretation of CMA findings remains a limiting factor hampering the selection of truly causative variants. Generally, the chromosomal imbalances associated with well-established microdeletion/microduplication syndromes are not a matter of concern, while abnormalities identified in genomic regions that have not been associated with human diseases yet might present some difficulties.

During 4 years, 1191 CMA analyses were performed in our department, in 1003 cases as a first-line cytogenetic test. Chromosomal aberrations were identified in 25% of our patients, and 41% of the findings were considered causative or likely causative of the phenotype based on the recommendations provided by the International Standard Cytogenomic Array Consortium and American College of Medical Genetics (Miller et al. 2010; Kearney et al. 2011b). The overall diagnostic yield of clinically relevant findings (11%) was in concordance with previous reports (Sagoo et al. 2009; Miller et al. 2010; Beaudet 2013). The diagnostic yields for 2009–2010 and 2011–2012 were 15% and 10%, respectively. This is due to the fact that patients analyzed by CMA during 2009–2010 were very carefully selected by clinical geneticists, whereas the group of patients analyzed in 2011–2012 was more heterogeneous and also included patients with nonspecific milder phenotypes. At the same time, the proportion of VUCS was quite high (41% of all findings). It seems that our ability to detect CNVs has far outpaced our ability to understand their role in disease (Coughlin et al. 2012). Actually, VUCS require additional time-consuming, expensive confirmation tests and recurrent counseling, which all causes further costs for health insurance funds. Due to that, we limited the amount of confirmation studies. In addition, although a new and not yet understood finding may help in the future to understand the cause of disease, currently VUCS often cause stress and uncertainty for parents and patients.

Among the primary tests recommended for estimation of the VUCS's pathogenicity are inheritance studies, although it is often imprudent to attribute clinical significance based on the inheritance pattern of a CNV in a single family (Kearney et al. 2011b). In this study, the inheritance analyses were completed for about half of the deletions/duplications with uncertain clinical relevance, and only three imbalances out of 28 appeared to be de novo. Still, the pathogenicity of inherited CNVs cannot be excluded before more information on those genomic regions is available, as a growing number of recurrent CNVs display variable penetrance or expressivity and may confer susceptibility or risk, rather than be directly causative (Cooper et al. 2011; Howell et al. 2013). In addition, it should be kept in mind that parentally segregated CNVs could contribute to proband's phenotype through epigenetic effects, or by unmasking a recessive mutation on a nondeleted allele (Kearney et al. 2011b; Battaglia et al. 2013). The situation with de novo mutations is also not so straightforward. Although the “de novo” status is usually taken as evidence supporting pathogenicity, it has been demonstrated that many regions of the genome have significantly elevated mutation rates, and some CNVs may indeed be de novo mutations yet have no clinical significance (Bradley et al. 2010).

In addition to detecting CNVs, SNP microarrays can also identify copy-number-neutral events such as LCSH. The presence of multiple LCSH distributed across different chromosomes can indicate a familial relationship between the proband's parents and usually represents an unexpected finding. Four such cases were identified among our patients, with the percentage of the autosomal genome, that is, IBD varying from 4% to 22%; however, these percentages are clearly underestimated as only those segments of homozygosity meeting a threshold of 5 Mb set by our laboratory were included for calculation. Generally, a high percentage (>10%) would be a sign of a close parental relationship, and in this case, laboratory report should indicate that the results could be associated with possible consanguinity. However, the specific familial relationship or degree of parental relatedness cannot always be extrapolated from the inbreeding coefficient; therefore, speculations of a specific relationship must be avoided in laboratory reports (Rehder et al. 2013).

A large region of homozygosity observed on a single chromosome may be indicative of UPD. Four cases of UPD associated with patients’ clinical phenotypes were found, including three mosaic cases: 4q31.3-q35.2 (50%), 11p15.5-p15.4 (50%) – Beckwith–Wiedemann syndrome, UPD 14, and maternal UPD 15 (50%) – Prader–Willi syndrome. However, single LCSH events, especially smaller ones, are generally difficult to interpret. Most detected LCSH likely represent regions of suppressed recombination or linkage disequilibrium, although potentially they may be associated with recessive diseases. The genomic content of the region should be evaluated in regard of patient's clinical problems, which assumes a close collaboration between clinical and laboratory staff members (Howell et al. 2013). Subsequently, the confirmation of the pathogenicity of such chromosomal abnormalities requires sequencing of the candidate gene of interest. Nevertheless, most of LCSH detected in our patients were classified as VUCS, because it was impossible to establish a link between phenotype and CMA finding.

Thus, the referring pediatrician or neurologist should be aware of the possibility that CMA provides results which are often random or difficult to interpret. Open-access databases of clinically relevant (e.g., DECIPHER) as well as nonpathogenic CNVs (e.g., DGV) are extremely helpful for interpreting CMA results, therefore, it is very important that as many centers as possible contribute to the development and completion of these resources. It should be mentioned that due to expanding knowledge, including first of all the addition of new entries to the publicly available databases during 2009–2012, a significantly large portion (61 of 351) of chromosomal imbalances reported to our patients can now be recategorized as benign or likely benign.

By turn, the issue every diagnostic laboratory should consider is the choice of array platform, which has to present a balance between sensitivity and specificity. Obviously, there is no need for maximum resolution in a genome-wide clinical test, as this is accompanied with an increase in the number of findings with uncertain clinical significance. The resolution of ∼400 kb throughout the genome with probe enrichment in regions of known clinical relevance is recommended and enables to reliably identify all known recurrent microdeletion and microduplication syndromes and most nonrecurrent imbalances that are unequivocally pathogenic (Miller et al. 2010; Kearney et al. 2011a). In addition, one can choose between two possible options: SNP-arrays and array-based comparative genomic hybridization (aCGH), which both are highly efficient tools used in research as well as in clinics. Both microarray types are suitable for detecting DNA copy-number changes and they are also capable of identifying low-level mosaicism. However, a meiotic or mitotic origin of the latter can only be distinguished using SNP-arrays. Furthermore, the genotype information provided by SNP-arrays allows the recognition of copy-number-neutral events, such as LCSH. It should be discussed whether a particular diagnostic center is interested in detection of such kind of aberrations, as usually they represent an issue of concern in regard of interpretation and counseling. Also, the genotype data obtained by SNP-arrays are useful when parental origin of an aberration is crucial and is necessary to be determined, although in this case a trio (a patient and both parents) should be analyzed. When choosing the array platform, the throughput numbers should also be considered. We mainly use HumanCytoSNP-12 Beadchips, which allow simultaneous analysis of 12 patients. However, it may be a problem in smaller centers to have 12 DNA samples readily available, which might be an issue especially in urgent prenatal testing.

.The proper interpretation of CMA results is particularly challenging in prenatal testing, where limited information on the fetal phenotype is accompanied with time pressure. Currently, CMA is mainly applied in parallel with traditional cytogenetic analyses, and a number of reports comparing the diagnostic efficacy of these approaches have been published (Hillman et al. 2011, 2013; Wapner et al. 2012; Fiorentino et al. 2013). However, the CMA application in prenatal diagnosis remains controversial. The American College of Obstetrics and Gynecology and the Italian Society of Human Genetics recommend that karyotyping remains the principle cytogenetic tool in prenatal diagnosis and microarrays should be used as an additional test in case of abnormal ultrasound finding; while some authors propose the method to be used as a first-tier test for high-risk pregnancies, because CMA is capable of identifying nearly all aberrations seen on karyotyping and offers a higher detection rate as compared with the latter (ACOG 2009; Novelli et al. 2012; Wapner et al. 2012; Yatsenko et al. 2013). In our prenatal cohort of 52 high-risk pregnancies and eight fetuses tested after the termination of pregnancy, CMA was mostly used in conjunction with conventional karyotyping. As expected, the unbalanced changes observed on G-banding were also seen by CMA, while balanced rearrangements remained undetected. Low-level mosaic trisomy 7 (∼10%) was also identified by both CMA and karyotyping. In addition, CMA identified multiple LCSH in two cases and a small pathogenic deletion that would be missed by traditional methods. Because of the relatively small prenatal cohort, we avoid making any conclusions about applying CMA as a first-line test in prenatal diagnosis. Obviously, with the increase in our knowledge and ability to explain any microarray finding, CMA will replace karyotyping, as it has already happened in pediatric populations. However, due to its shorter turnover, CMA could currently be recommended as a primary cytogenetic test when time is a limiting factor.

Despite the great utility of CMA in clinical practice, chromosome analysis and FISH still remain useful tools for leftacterization of structural aberrations, because important disease mechanisms may go undiagnosed and may be underestimated if only CMA is performed. In addition, balanced translocations or inversions, which are present in 0.78% of patients with idiopathic ID and in 0.08–0.09% of prenatal diagnostic samples, are not detectable by CMA (Giardino et al. 2009; Hochstenbach et al. 2009). However, apparently balanced de novo rearrangements are associated with a 6.7% risk of serious congenital anomaly (Warburton 1991). Depending on the microarray platform, low-level mosaic cases could also be missed. Therefore, a full-chromosome analysis may be considered for patients with a normal CMA result and MCA, dysmorphic features, and/or ID reminiscent of a chromosomal syndrome or clinical manifestations indicative of potential mosaicism (Coughlin et al. 2012).

In summary, our experience demonstrates once more that CMA is a useful cytogenetic tool for detecting the genomic reason underlying DD/ID, ASD, and/or MCA phenotypes in a significant portion of the patients. However, close cooperation between clinicians and cytogeneticists, as well as data sharing with colleagues are the cornerstones of successful CMA application in clinical practice.

## Web Resources

Database of Genomic Variants: http://projects.tcag.ca/variation/. DECIPHER: https://decipher.sanger.ac.uk/application/. Ensembl: http://www.ensembl.org/index.html. Online Mendelian Inheritance in Man (OMIM): http://www.ncbi.nlm.nih.gov/omim. UCSC Genome Browser: http://genome.ucsc.edu.
